# Sensitivity Enhancement in Surface Plasmon Resonance Biochemical Sensor Based on Transition Metal Dichalcogenides/Graphene Heterostructure

**DOI:** 10.3390/s18072056

**Published:** 2018-06-27

**Authors:** Xiang Zhao, Tianye Huang, Perry Shum Ping, Xu Wu, Pan Huang, Jianxing Pan, Yiheng Wu, Zhuo Cheng

**Affiliations:** 1 School of Mechanical Engineering and Electronic Information, China University of Geosciences (Wuhan), Wuhan 430074, China; zhaoxiang@cug.edu.cn (X.Z.); wuxu101393@cug.edu.cn (X.W.); huang_pan@cug.edu.cn (P.H.); jianxing_pan@cug.edu.cn (J.P.); wuyiheng@cug.edu.cn (Y.W.); chengzhuo@cug.edu.cn (Z.C.); 2 Center of Fiber Technology, School of Electrical and Electronic Engineering, Nanyang Technological University, Singapore 639798, Singapore; EPShum@ntu.edu.sg

**Keywords:** surface plasmon resonance, transition metal dichalcogenides, angular sensitivity, differential phase

## Abstract

In this work, a surface plasmon resonance (SPR) biosensor based on two-dimensional transition metal dichalcogenides (TMDCs) is proposed to improve the biosensor’s sensitivity. In this sensor, different kinds of two-dimensional TMDCs are coated on both surfaces of metal film. By optimizing the structural parameters, the angular sensitivity can reach as high as 315.5 Deg/RIU with 7-layers WS_2_ and 36 nm Al thin film, which is 3.3 times of the conventional structure based on single Al thin film. We also obtain maximum phase sensitivity (3.85 × 10^6^ Deg/RIU) with bilayer WS_2_ and 35 nm Al thin film. The phase sensitivity can be further improved by employing Ag and removing air layer. The proposed configuration is of great potential for biochemical sensing.

## 1. Introduction

Surface plasmon resonance (SPR) is an optical phenomenon which occurs at the metal-dielectric surface. When light reflects at a SPR angle, free electrons on the metal surface can resonate and absorb light energy, consequently leading to a drastic attenuation of reflected light [1,2]. The SPR condition is sensitive to the environment variations and can be utilized as sensors. The biological molecules interactions in the sensing medium are detected by observing the refractive index changes of the sensor region. Due to advantages such as convenient detection, high sensitivity, real-time measurement, SPR sensors have been used to detect and analyze various biological molecules, such as proteins, nucleic acids and viruses, and have a broad prospect in practical applications [3,4,5,6]. Sensitivity is one of the most important aspects for biological sensing in particular, and how to enhance the sensitivity becomes a research hotspot for SPR biosensors.

Recently, 2D materials such as graphene and transition metal dichalcogenides (TMDCs) have are well-known for their use in constructing SPR sensors due to their unique electrical and optical properties [7]. This is because firstly, the high real part of the dielectric constant allows them to help metal absorb light energy [8]. Secondly, some features such as high surface to volume ratio and tunable biocompatibility can help the biosensor obtain sensitivity enhancements [9]. Finally, when coating these materials on the metal film, they can also protect the metal from oxidation as protective layers [10,11]. Based on these advantages, various 2D-material-assisted SPR sensors are proposed and investigated. Graphene has been proposed for the enhancement of the sensitivity [12]. Zeng et al. presented a highly sensitive SPR biosensor based on graphene-MoS_2_ hybrid nanostructures to enhance its sensitivity [13]. Air layer and graphene sheet for sensitivity enhancement was analyzed in [14]. Other TMDCs like WS_2_, MoSe_2_ and WSe_2_ are combined with silicon to enhance the sensitivity [15]. Wu et al. proposed a SPR biochemical sensor with heterostructures of few-layer BP and 2D materials (graphene/MoS_2_/WS_2_/MoSe_2_/WSe_2_) [16]. According to the previous work, it is found that the sensor performances are highly related to the structures and functional materials. To further enhance the sensitivity, both of these two aspects should be properly optimized.

In this paper, SPR sensor constructed by TMDCs/metal/TMDCs/graphene heterostructure is used for both angular and phase sensitivity enhancement. By coating different 2D TMDCs (MoS_2_/MoSe_2_/WS_2_/WSe_2_) at both sides of the metal, the sensitivity of the proposed sensor can be improved by the enhancement of light-material interaction. Angular sensitivity as high as 315.5 Deg/RIU which is nearly 3 times that of conventional configurations can be obtained. Furthermore, the proposed SPR configuration is suitable for phase detection as well and a pronounced phase sensitivity up to 3.85 × 10^6^ Deg/RIU is predicted.

## 2. Sensor Configuration and Theoretical Model

**Figure 1:**
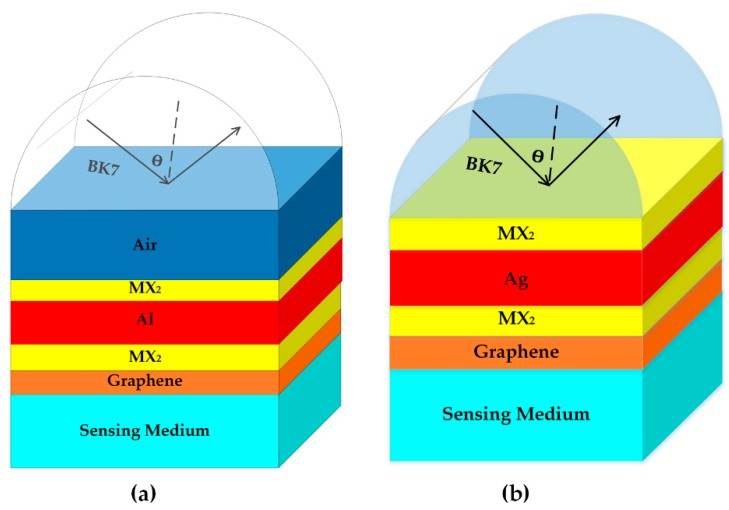
Schematic diagram of the SPR biosensor for (**a**) angle-sensitivity enhancement and (**b**) phase-sensitivity enhancement.

The schematic diagram of the proposed SPR biosensor is shown in Figure 1a, the configuration contains seven layers and the operation wavelength is 633 nm which is popular for SPR applications [13,14,17]. BK7 glass with refractive index of *n*_p_ = 1.5151 acts as the coupling prism [18]. The refractive index of the air layer is fixed at 1 with thickness of 35 nm. The metal employed in this configuration is Al with dielectric constant of −34.2574 + 0.9108i [19]. Various TMDCs, represented by MX_2_, are coated at both sides of Al thin film, the thickness and refractive index of TMDCs at 633 nm are shown in Table 1 [20,21]. The graphene layer is coated on the MX_2_/Al/MX_2_ hybrid structure as the biomolecular recognition element and the refractive index of graphene is given as [22]:

(1) $$n_{G}=3.0+\frac{iC_{1}\lambda }{3}$$

where *λ* is the wavelength and *C*_1_ = 5.446 μm^−1^. The thickness of the monolayer graphene is 0.34 nm. The refractive index of the sensing medium is given as *n*_s_ = 1.33 + ∆*n*, where ∆*n* is the index change of the sensing medium.

In this paper, for the SPR curve calculation and sensing performance analysis, the transfer matrix method (TMM) is employed [23]. In the proposed structure, the thickness, the refractive index, and the dielectric constant of each layer are defined as *d*_k_, *n*_k_ and *ε*_k_, respectively. The incident angle corresponding to the minimum reflectance is called resonance angle and the angular sensitivity is calculated by probing the spectral shifts of the resonance angle [3] and defined as *S*_A_ = ∆*θ*_res_/∆*n* [24], where ∆*θ*_res_ represents the change of resonance angle. Furthermore, we also discuss phase sensitivity which is defined as *S*_p_ = ∆*φ*/∆*n* [13], where ∆*φ* is the differential phase changes corresponding to ∆*n*.

## 3. Results and Discussions

**Figure 2:**
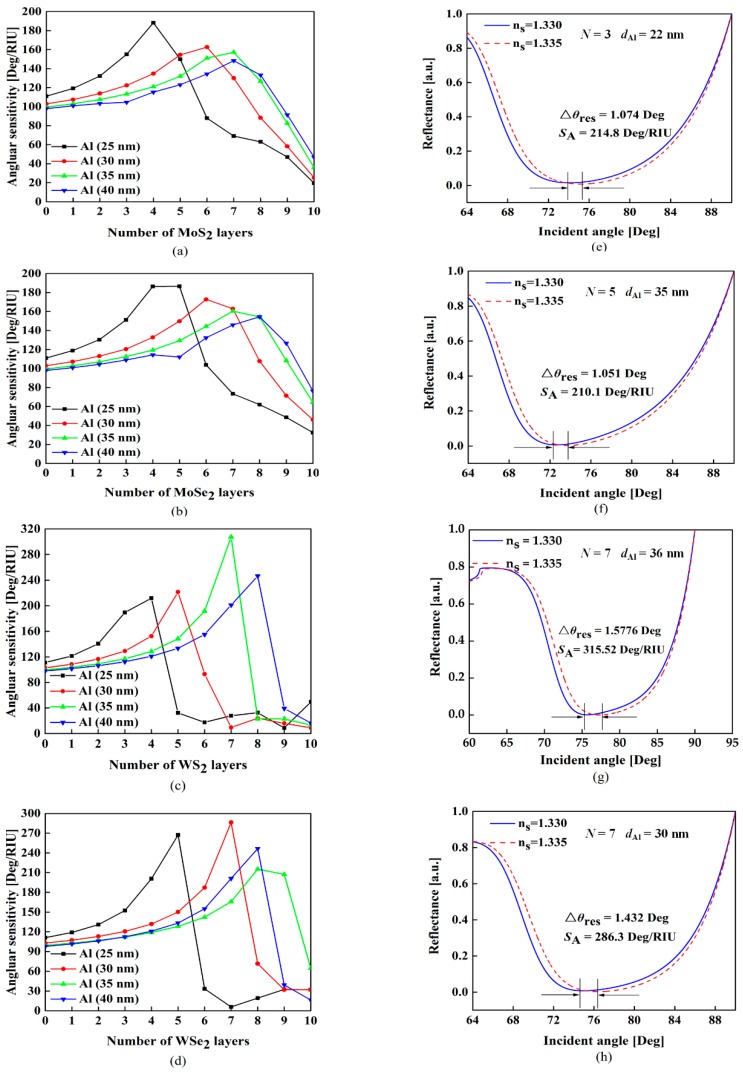
(**a**–**d**) Variation of the angular sensitivity as a function of the number of MX_2_ layers *N* with various thickness of Al thin film *d*_Al_ and (**e**–**h**) the reflection spectra with different TMDCs materials under optimal structures.

In order to obtain the optimal angular sensitivity for the proposed configuration, we firstly calculate the angular sensitivity with various number of MX_2_ layers and Al thickness. It should be noted that in this calculation, the MX_2_ layers at both sides are changed simultaneously. As shown in Figure 2a–d, when the refractive index of sensing medium changes from 1.330 to 1.335 (Δ*n* = 0.005), the SPR curves shows three important features enumerated below.

(1) When the thickness of Al thin film is fixed, the sensitivity increases with more MX_2_ layers mainly due to the enhanced light energy absorption. However, it will decrease rapidly when the number of MX_2_ layers exceeds the optimal number which is defined as the number of MX_2_ layers with the highest sensitivity.
(2) The optimal numbers of MX_2_ layers will increase when the thickness of Al increases.
(3) With the same thickness of Al thin film, the enhancement effect offered by different kinds of MX_2_ are not the same.

It is known that the improvement effect caused by TMDCs is related to their dielectric constants. As illustrated in Table 1, MoS_2_ has a larger real part of the dielectric constant than others, which means its absorption ability is stronger [15]. Nevertheless, the ability to absorb light is not the only crucial factor to affect sensitivity; electron energy loss related to the imaginary part of the dielectric constant can lead to a counteraction [13]. Comparing to other TMDCs, the WS_2_ layers have much lower energy loss because of their small imaginary part of the dielectric constant. According to the conditions above, the optimized parameters and the corresponding angular sensitivity for different TMDCs are summarized in Table 2. The highest sensitivities can achieve 214.8 Deg/RIU, 210.1 Deg/RIU, 315.5 Deg/RIU and 286.3 Deg/RIU for the structures containing 3-layer MoS_2_ with 22 nm Al film, 4-layer MoSe_2_ with 24 nm Al film, 7-layer WS_2_ with 36 nm Al film and 7-layer WSe_2_ with 30 nm Al film, respectively. The corresponding SPR curves are shown in Figure 2e–h.

**Figure 3:**
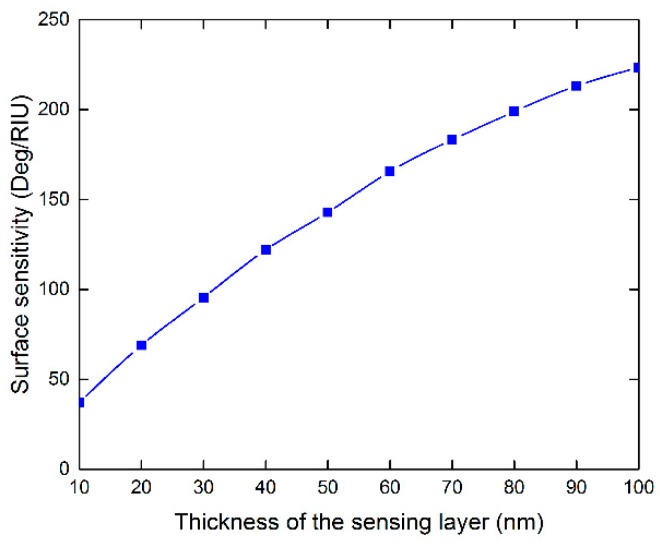
The change in surface sensitivity as the function of the thickness of biomolecules on the surface.

It should be noted that the intensity of reflection light is very weak at resonance. In order to effectively measure the resonance shift, it is necessary to increase the incident power or adopt detectors with high sensitivity. For example, assuming 10 mW incident light and 0.1 deg angular resolution [25], the reflected power at the SPR dip is estimated to be 6.5 μW and the reflected power variation between the resonance angle and its nearest measurable neighbor is 0.32 μW, which can be easily detected by commercially-available photodiodes at a visible wavelength [26]. Also note that, in our calculation, the sensing medium is set to be homogeneous in order to make fair comparison with previously works [12,13,14,15,16]. In fact, when the SPR biosensors are used for detecting cells with size of several microns [27], a homogeneous sensing medium layer is reasonable. To further investigate the surface sensitivity, the index change caused by the sensing target is applied with finite thickness. Under the optimal condition for WS_2_, the sensitivities with different sensing layer thickness are shown in Figure 3. It is shown that the surface sensitivity increases with thicker sensing layer. Even with 10 nm thickness, the surface sensitivity can be still as high as 37 Deg/RIU.

**Figure 4:**
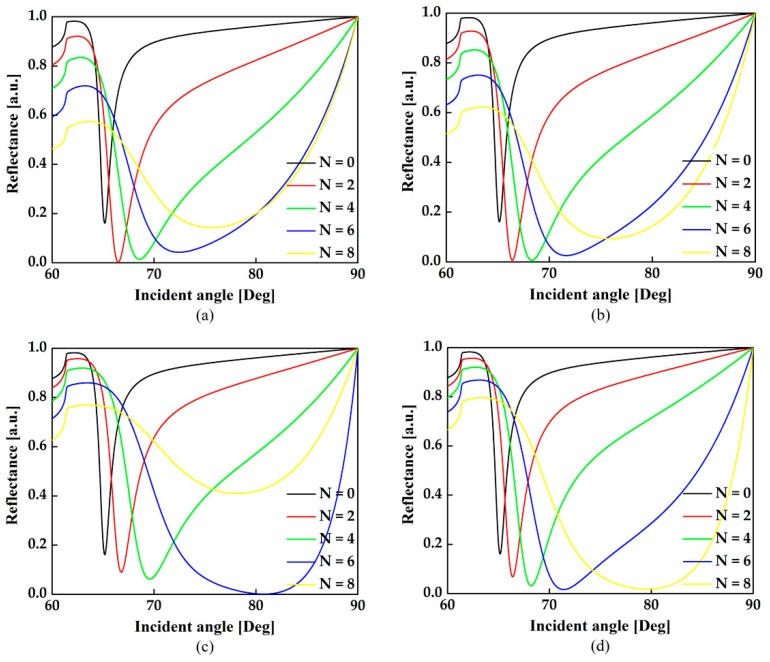
Variation of the reflectance respect to the different number of (**a**) MoS_2_, (**b**) WS_2_, (**c**) MoSe_2_ and (**d**) WSe_2_ layers with the thickness of Al thin film is fixed at 30 nm and the refractive index of sensing medium ∆*n* = 1.3300.

To further illustrate the contribution of MX_2_ on sensitivity, we have drawn the variation of the reflectance with incident angle varying from 60 Deg to 90 Deg for the different number of layers when the thickness of Al thin film is fixed at 30 nm and *n*_s_ = 1.3300 in Figure 4a–d. It is indicated that the minimum reflectivity, representing the capability of light energy absorption [15], approaches zero firstly when the number of MX_2_ layers increase, which means the contribution of light energy absorption exceeds the electron energy loss. Meanwhile, the full width at half maximum (FWHM) becomes broader caused by electron energy loss of MX_2_ layers [13]. With the further increase of the MX_2_ layers, the FWHM keeps getting broader and the minimum reflectivity begins to diverge from zero. This is because the SPR process must satisfy the energy conservation *T* + *R* + *A* = 1, where *T*, *R* and *A* denote the transmission, reflection and absorption, respectively. Under SPR condition, since the total internal reflection is fulfilled, the transmission *T* is close to 0. When the number of TMDCs layers is insufficient, the absorbed light energy is not able to promote a strong SPR excitation. Therefore, increasing the TMDCs layers can enhance the light absorption, resulting in higher sensitivity. In this condition, the absorption *A* is enhanced while the reflection *R* is reduced. However, the absorption enhancement will be saturated due to the electron energy loss when further adding TMDCs layers. In this condition, the absorption *A* is degraded while the reflection *R* is increased.

**Figure 5:**
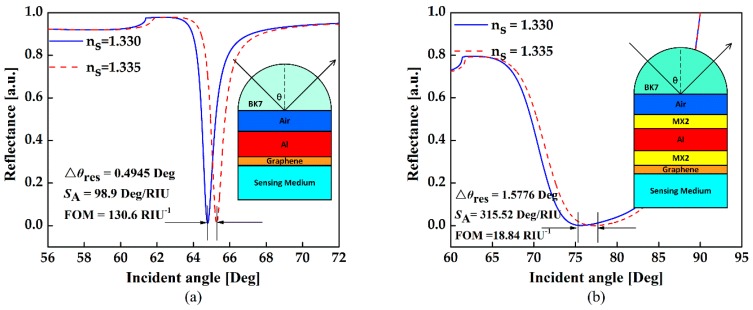
Variation of the reflectance with respect to the incident angle for (**a**) the conventional biochemical sensor based on single Al film, and (**b**) the proposed configuration based on optimal angle sensitivity.

**Figure 6:**
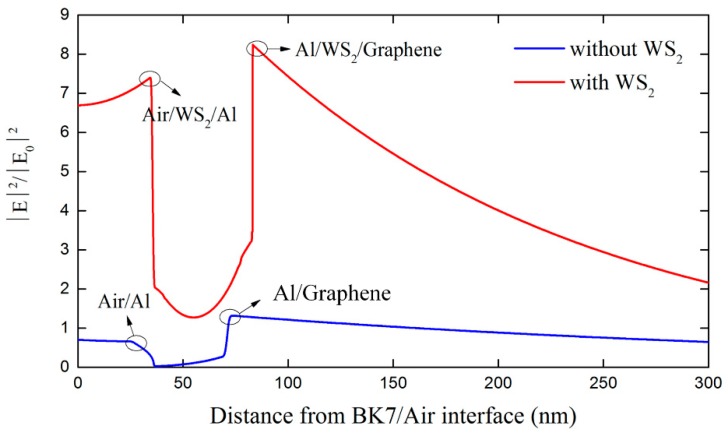
The field distributions along the direction perpendicular to the prism for the proposed configuration with seven WS_2_ layers and the conventional configuration without WS_2_ layers.

The sensitivity of conventional structure based on single Al thin film is not high enough since the metallic layer cannot absorb enough light energy to excite a strong SPR. However, TMDCs has a larger real part of dielectric constant, which benefits SPR excitation [15]. Figure 5 shows the configuration and reflectance curves for the conventional SPR sensors and the proposed one with optimal parameters. Prominent sensitivity improvement up to 3.3 times can be observed in the WS_2_-assisted configuration. Figure 6 plots the electric field distributions in these two structures. There is a stronger field enhancement in the WS_2_-assisted structure compared to the conventional one, which further verifies the positive contribution provided by the TMDCs. In addition to sensitivity, figure of merit (FOM) is also one of the important aspects that affects the sensing performance. According to our calculation, the structure without TMDCs demonstrates the highest FOM. This is because the energy loss induced by the TMDCs will broaden FWHM. As we know, this phenomenon is also reported in other works of TMDCs-based SPR sensors [12,13,14,15,16]. Therefore, introducing TMDCs into the SPR sensor will contribute to sensitivity enhancement but not FOM enhancement.

**Figure 7:**
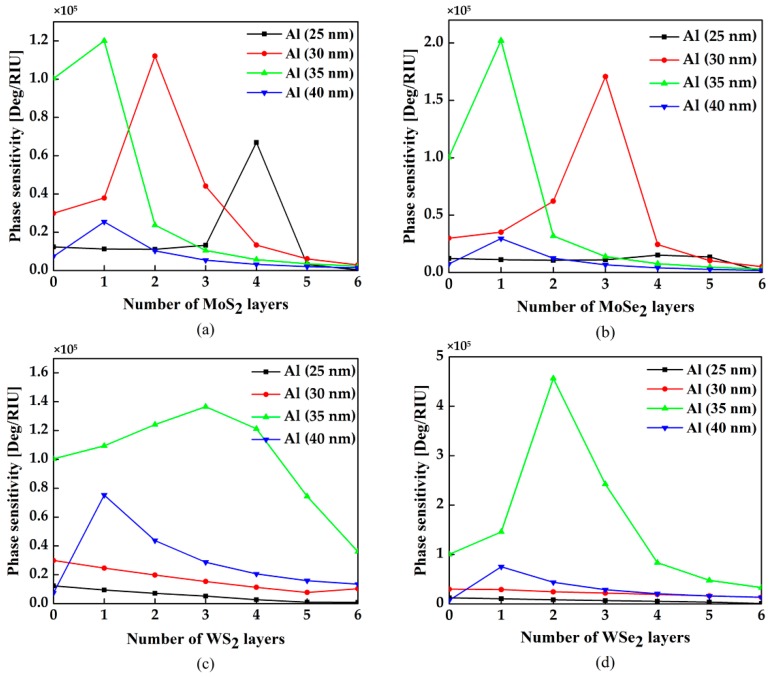
Variation of the angular sensitivity as a function of number of MX_2_ layers with various thickness of Al thin film, (**a**) MoS_2_, (**b**) MoSe_2_, (**c**) WS_2_, and (**d**) WSe_2_.

Besides angular sensitivity, the differential phase change between p-polarized and s-polarized reflective wave is another approach to detect the analyte [28,29,30]. The variation of the phase sensitivity with respect to the different number of MX_2_ and thickness are showed in Figure 7a–d. In the structure showing in Figure 1a, we can obtain the highest sensitivity of 1.12 × 10^5^ Deg/RIU for bilayer MoS_2_ with 30 nm Al film, 2.02 × 10^5^ Deg/RIU for monolayer MoSe_2_ with 35 nm Al film, 1.37 × 10^5^ Deg/RIU for 3-layer WS_2_ with 35 nm Al film and 4.56 × 10^5^ Deg/RIU for bilayer WSe_2_ with 35 nm Al film, respectively. Comparing with angular sensitivity, the optimal number of MX_2_ layers for phase sensitivity is less.

To further improve the phase sensitivity, we propose another SPR biosensor based on Kretschmann configuration, as shown in Figure 1b. In this sensor, Ag is used to replace Al and the air layer is removed. The optimal conditions of Ag thickness and MX_2_ layers are summarized in Table 3. The best phase sensitivity as high as 3.85 × 10^6^ Deg/RIU can be achieved with monolayer WS_2_ and 46 nm Ag film.

For comparison, the performances of previously reported 2D-material-assisted SPR sensors are summarized in Table 4. Significant enhancements on both angular sensitivity and phase sensitivity can be obtained in the proposed sensors.

## 4. Conclusions

In this paper, SPR biosensors by using 2D TMDCs are proposed to enhance the sensitivity. In such sensors, the functional materials are coated on both sides of the metal layer and the impacts of material type, layer number, and metal thickness on the sensing performance are investigated and analyzed in detail. The results show that the angular sensitivity and phase sensitivity can reach as high as 315.5 Deg/RIU with 7-layers WS_2_ and 3.85 × 10^6^ Deg/RIU with 1-layers WS_2_, respectively. The proposed configuration can be promising a candidate for high performance biosensing.

## Figures and Tables

**Table 1:** The thickness, refractive index and optical constants of different MX_2_ at *λ* = 633 nm.

Type of TMDC	Thickness of Monolayer (nm)	Refractive Index	Dielectric Constant
MoS_2_	0.65	5.0805 + 1.1723i	24.4368 + 11.9121i
MoSe_2_	0.70	4.6226 + 1.0063i	20.3560 + 9.3039i
WS_2_	0.80	4.8937 + 0.3124i	23.8511 + 3.0578i
WSe_2_	0.70	4.5501 + 0.4332i	20.5156 + 3.9423i

**Table 2:** Optimized values of thickness of gold and the number of MoS_2_ layers with corresponding change in angular sensitivity.

Type of TMDC	Optimal Thickness of Al (nm)	Optimal Number of TMDC Layers	Angular Sensitivity (Δ*n* = 0.005)
MoS_2_	22	3	214.8 Deg/RIU
MoSe_2_	24	4	210.1 Deg/RIU
WS_2_	36	7	315.5 Deg/RIU
WSe_2_	30	7	286.3 Deg/RIU

**Table 3:** Variation of the angular sensitivity as a function of number of MX_2_ layers with various thickness of Al thin film.

Type of TMDC	Optimal Thickness of Al (nm)	Optimal Number of TMDC Layers	Angular Sensitivity (Δ*n* = 0.005)
MoS_2_	40	2	6.32 × 10^5^ Deg/RIU
MoSe_2_	40	2	1.54 × 10^5^ Deg/RIU
WS_2_	46	1	3.85 × 10^6^ Deg/RIU
WSe_2_	44	1	4.57 × 10^5^ Deg/RIU

**Table 4:** Comparison with the formerly reported 2D-material-assisted SPR sensors.

2D Material	Metal	Angular Sensitivity	Phase Sensitivity	References
Graphene	Au	134.6 Deg/RIU	-	[12]
Graphene and MoS_2_	Al	190.4 Deg/RIU	-	[13]
Graphene and MoS_2_	Au	-	8.19 × 10^4^ Deg/RIU	[14]
WS_2_	Au	155.7 Deg/RIU	-	[15]
WSe_2_	Au	-	1.20 × 10^6^ Deg/RIU	[15]
BP and TMDCs/graphene	Ag	279.0 Deg/RIU	6.75 × 10^3^ Deg/RIU	[16]
WS_2_ and graphene	Al	315.5 Deg/RIU	-	This work
WS_2_ and graphene	Ag	-	3.85 × 10^6^ Deg/RIU	This work
